# Machine Learning Detects Symptomatic Plaques in Patients With Carotid Atherosclerosis on CT Angiography

**DOI:** 10.1161/CIRCIMAGING.123.016274

**Published:** 2024-06-18

**Authors:** Francesco Pisu, Brady J. Williamson, Valentina Nardi, Kosmas I. Paraskevas, Josep Puig, Achala Vagal, Gianluca de Rubeis, Michele Porcu, Riccardo Cau, John C. Benson, Antonella Balestrieri, Giuseppe Lanzino, Jasjit S. Suri, Abdelkader Mahammedi, Luca Saba

**Affiliations:** 1 Department of Radiology, Azienda Ospedaliero-Universitaria, Monserrato (Cagliari), Italy (F.P., M.P., R.C., A.B., L.S.).; 2 Department of Radiology, University of Cincinnati, Cincinnati, OH (B.J.W., A.V., A.M.).; 3 Department of Radiology, Mayo Clinic, Rochester, MN (V.N., J.C.B., G.L.).; 4 Stroke Diagnosis and Monitoring Division, Atheropoint LLC, Roseville, CA (J.S.S.).; 5 Department of Vascular Surgery, Central Clinic of Athens, Athens, Greece (K.I.P.).; 6 Department of Radiology (IDI), Hospital Universitari de Girona, Girona, Spain (J.P.).; 7 UOC Neuroradiology Diagnostic and Interventional, San Camillo-Forlanini Hospital, Rome, Italy (G.R.).

**Keywords:** atherosclerosis, angiography, calibration, carotid stenosis, hemorrhage

## Abstract

**BACKGROUND:**

This study aimed to develop and validate a computed tomography angiography based machine learning model that uses plaque composition data and degree of carotid stenosis to detect symptomatic carotid plaques in patients with carotid atherosclerosis.

**METHODS:**

The machine learning based model was trained using degree of stenosis and the volumes of 13 computed tomography angiography derived intracarotid plaque subcomponents (eg, lipid, intraplaque hemorrhage, calcium) to identify plaques associated with cerebrovascular events. The model was internally validated through repeated 10-fold cross-validation and tested on a dedicated testing cohort according to discrimination and calibration.

**RESULTS:**

This retrospective, single-center study evaluated computed tomography angiography scans of 268 patients with both symptomatic and asymptomatic carotid atherosclerosis (163 for the derivation set and 106 for the testing set) performed between March 2013 and October 2019. The area-under-receiver-operating characteristics curve by machine learning on the testing cohort (0.89) was significantly higher than the areas under the curve of traditional logit analysis based on the degree of stenosis (0.51, *P*<0.001), presence of intraplaque hemorrhage (0.69, *P*<0.001), and plaque composition (0.78, *P*<0.001), respectively. Comparable performance was obtained on internal validation. The identified plaque components and associated cutoff values that were significantly associated with a higher likelihood of symptomatic status after adjustment were the ratio of intraplaque hemorrhage to lipid volume (≥50%, 38.5 [10.1–205.1]; odds ratio, 95% CI) and percentage of intraplaque hemorrhage volume (≥10%, 18.5 [5.7–69.4]; odds ratio, 95% CI).

**CONCLUSIONS:**

This study presented an interpretable machine learning model that accurately identifies symptomatic carotid plaques using computed tomography angiography derived plaque composition features, aiding clinical decision-making.

**CLINICAL PERSPECTIVE**

Computed tomography angiography of the carotid arteries is an essential diagnostic tool in the assessment of patients presenting with cerebrovascular symptoms. Employing a standardized protocol, our investigation explored the composition of carotid artery plaques, discerning between key subcomponents including calcium, lipid, and intraplaque hemorrhage. Using the volumes of such subcomponents to develop an interpretable machine learning model, we were able to successfully differentiate symptomatic from asymptomatic plaques. We found that the ratio of intraplaque hemorrhage to lipid volume and the proportion of intraplaque hemorrhage to total plaque volume were key indicators of symptomatic plaques. Our model’s intelligibility enables clinicians to understand how predictions are generated, which in turn improves their ability to assess risk. For a given carotid plaque, it first assigns a symptomatic likelihood to each individual component, which is then added up to determine the overall likelihood of a plaque being symptomatic. This straightforward approach gives clinicians a clearer picture of what factors contribute to plaque instability and potential cerebrovascular risk. This marks a step towards automated artificial intelligence-based solutions for predicting future cerebrovascular events based solely on plaque morphology.

Stroke is a common cause of death and severe disability worldwide, and a significant proportion of ischemic stroke is related to carotid artery atherosclerosis.^1,2^ Current guidelines for stroke prediction are primarily based on the degree of stenosis.^1^ However, recent literature have shown that plaque structure and composition play a fundamental role in plaque vulnerability or stability.^3–7^ Previous studies have demonstrated that morphological and composition differences between plaques can predict the occurrence of ischemic strokes.^8–11^ Investigation of these unique biomarkers and their predictive value is an area of active research. Currently, computed tomography angiography (CTA) is widely used in the noninvasive assessment of carotid atherosclerosis.^7^ Recent technological advancements in CTA allow the quantification and characterization of atherosclerotic carotid plaque.

Indeed, several volumetric measures characterizing the anatomic features of the plaque can be obtained using different HU cutoffs,^2^ namely fibrous tissues, calcium tissues, lipid tissues as well as intraplaque hemorrhage (IPH).^2,8^ Among the multiple parameters that have been indicated as responsible for an increased vulnerability, IPH has been identified as a strong risk factor of cerebrovascular events (CVE).^2,8,12,13^

Machine learning (ML), a subfield of artificial intelligence, has gained significant interest lately due to its ability to automatically learn complex patterns in existing data, that are undetectable with conventional statistical methods, and make predictions based on unseen data.^14^ However, the clinical application of ML models has suffered from the black box problem of interpretability and explainability.^15^ Hence, the development of interpretable ML solutions is essential for enabling clinicians to make confident decisions.^16^ The aim of this study was to derive an interpretable ML model that used data of plaque composition derived from CTA of carotid arteries to detect symptomatic carotid plaques. Furthermore, the study aimed to assess the most significant predictors and investigate potential nonlinear associations with symptomatic status using ML techniques.

## METHODS

The data collection and protocols used in this study were approved by the institutional review board, and individual patient consent was waived because of the retrospective nature of the study. This study was designed, and article prepared according to the checklist for artificial intelligence in medical imaging (CLAIM, Table S1).

### Data Availability

The data underlying this study’s findings cannot be shared publicly as it would compromise the privacy of the participants. The data can be made available on reasonable written request to the corresponding author, L. Saba.

### Study Design and Participant Selection

This is a single-center, retrospective, diagnostic study of 163 patients with carotid atherosclerosis who underwent CTA of the carotid arteries from January 2013 to November 2017 at Azienda Ospedaliero-Universitaria di Cagliari. One hundred twenty-three of 163 patients have previously been reported in another study,^2^ we retrospectively collected data of 106 patients who underwent CTA between December 2017 and October 2019 at the same institution.

In cases where atherosclerotic plaque was present in both carotid arteries of a subject, both the left and right sides were considered. However, if only one side showed signs of pathology, the healthy side was not included.

We included adults (18+) with carotid atherosclerosis diseases. Carotid sonography was used as a screening tool to identify carotid stenosis according to the Mannheim consensus (ie, carotid wall thickness of >1.5 mm^17^). Symptomatic patients underwent CTA without prior ultrasound. Otherwise, CTA screening was performed if ultrasound showed pathological stenosis (according to the North American Symptomatic Carotid Endarterectomy Trial criteria^17^), features indicating plaque vulnerability (eg, irregular surface, ulcerations) or it was inconclusive due to anatomic conditions. Diabetes screening and presurgery analysis also constituted criteria for CTA.^12^

Patients were excluded if >1 week was passed between symptoms onset and imaging (average was 3 days, range was 0–7, where 0 was the same time of the event) or in case of doubt with other pathologies (hypoglycemia, migraine, postparoxysmal neurological dysfunction), concomitant intracranial pathology (brain tumor, abscess, encephalitis) or cardiac embolic source. We also excluded patients in case of symptomatic status due to posterior circle occlusion.

### Definition of Symptomatic

Neurological status at the time of CTA was defined as symptomatic or asymptomatic based on the TOAST (Trial of Org 10172 in Acute Stroke Treatment) criteria^18,19^ for individual arteries by the clinical care team. The readers of the CTA during data reassessment were blinded on the status of the patient. We classified as symptomatic those subjects who had plaque in the carotid artery ipsilateral to the CVE in the distribution of the anterior and middle cerebral arteries. Asymptomatic persons had no history of either remote or recent CVE at the time of the examination. The CVE was defined as carotid territory ischemic event (TIA or stroke) with symptoms like hemiparesis, dysarthria, dysphasia, or monocular blindness. Episodes of neurological dysfunction that lasted >24 hours were considered as stroke, otherwise TIA.^11^ All symptomatic patients had subtype 1 (ie, large-artery atherosclerosis) as the cause. Stroke causes other than large-artery atherosclerosis, as per the TOAST criteria, were excluded by the clinical care team through a comprehensive cardiovascular examination. This included 12-lead electrocardiography, 24-hour Holter electrocardiography, transthoracic echocardiography, and hematologic screening.

### CTA Technique

Data were obtained using a 16-detector row CT system (Brilliance; Philips Healthcare, Best, the Netherlands). Images were obtained from the aortic arch to the circle of Willis before and after the administration of contrast. For CTA, 80 mL of prewarmed contrast medium (Ultravist 370; iopromide; Bayer HealthCare, Berlin, Germany) was injected into a cubital vein using a power injector at a flow rate of 4–5 mL/s and a 16-gauge intravenous catheter followed by 30 mL of saline flush. CT scanning parameters included the following: section thickness = 0.6 mm, section interval = 0.3 mm, matrix size = 512×512 pixels, FOV = 14–19 cm. Images were reconstructed using a C-filter algorithm. The same CT scanner was used for all the imaging.

### Carotid Plaques Analysis

Two expert radiologists (L. Saba and A. Balestrieri) blinded to symptomatic status performed all HU measurements using a window/level setting of 850/300. To analyze the volume of plaques and their subcomponents, a semi-automated software (iNtuition; Terarecon, Foster City, CA) was used, which involved the delineation of the inner and outer wall boundaries of the vessel and the calculation of the volume of the plaque and its subcomponents based on the specific region of interest.^9,10^ To identify and classify the subcomponents of the plaque, attenuation values of all voxels within a volume were identified, and specific thresholds were used to classify the tissues according to the attenuation values. Through the use of specific thresholds, 5 tissue classes can be identified: lipid tissue (<60 HU), fibrous tissue (60–130 HU), calcium tissue (>130 HU) as suggested by de Weert et al,^20^ as well as IPH (<25 HU) and lipid—IPH (26–59 HU) which are subclasses of the lipid tissue category.^2,21^ In this regard, IPH and lipid-IPH are subclassifications of the lipid tissue class. Interobserver and intraobserver variability of the volumetric analysis was not measured in this study as it has already been done in previous investigations.^17,22^

### Machine Learning

Data on 14 variables were available for ML. They included presence of stenosis, intracarotid plaque and its subcomponents volumes derived from CTA scans (lipid, fibrous, calcium, IPH and lipid minus IPH) in both absolute and percentage values, and the ratio of IPH to lipid volume. See Table S2 for details. The plaque’s symptomatic status was the binary outcome, and models were trained to detect symptomatic plaques.

The ML methodology involved training and validating statistical and ML models on the derivation cohort with 10 repetitions of 10-fold CV. A separate cohort was used to test the ML model and determine the significance of predictors of symptomatic status and relative cutoffs. Finally, nonlinear associations between selected variables and the symptomatic status were investigated.

#### Model Building

The gradient-boosting generalized additive model (GB-GAM) was used to derive models for identifying symptomatic plaques. The GB-GAM is a highly intelligible model that learns a function for each individual feature using gradient boosting, and combines them to derive the likelihood of symptomatic status.^23^ The GB-GAM has been effectively used in other studies with cardiac data.^24,25^ In gradient boosting, a kind of supervised ensemble learning, weaker estimators are ensembled to produce a stronger estimator by sequential fitting and adjustment of weighting to account for misclassifications. For an unseen carotid artery, feature values are used to index learned functions, which in turn provide partial contributions that are summed up to predict the score of symptomatic status.

Four different models were derived using a GB-GAM with plaque composition data and presence of stenosis (ML), and 3 logistic regressors using presence of stenosis, presence of IPH, and plaque composition data, respectively. We refrained from performing hyperparameter tuning due to the small sample size and hence used settings derived from previous works instead.

#### Internal Validation and Dedicated Testing

Model derivation was conducted in a stratified 10-fold CV with 10 repetitions, which ensures robust performance when the sample size is small.^26^ Briefly, the data is divided into 10 equally sized subsets ensuring similar proportions of cases in each subset. Nine out of 10 sets were used for model derivation and the remaining set was used for validation. This protocol was applied 9 more times so that each set was used for validation exactly once. Overall, this scheme was repeated 10×, each time shuffling the dataset to better estimate the generalization error of the model.

Compared with the conventional holdout method, 10-fold CV allows to: (1) reduce the bias when quantifying predictive performance, (2) reduce the variance when estimating the generalization error, and (3) maximize the available data for training and testing by ensuring these are performed on nonoverlapping subsets, hence limiting the risk of overfitting.

Each model was used to predict the probability of symptomatic plaque for all arteries in each testing fold; these scores were pooled and used to quantify predictive performance and to compare models according to the area under the receiver-operating characteristics (AUROC) and area under the precision-recall (AUPR) curves.

Finally, the ML model was trained using all available data using the same methodology as used within CV and evaluated on a dedicated testing cohort.

To address potential bias arising from the underrepresentation of female patients in our datasets, we derived all models using a gender-balanced derivation set. These models were then employed to classify patients in the dedicated testing set, aiming to assess whether a gender-balanced model would exhibit fewer errors on female subjects compared with the model trained on the unbalanced derivation set. This approach was implemented to alleviate the minority representation of female subjects compared with male subjects. Additional details on the balancing technique can be found in the Supplemental Methods.

#### Importance of Variables and Contribution to Predictions

Features were ranked by average absolute impact on model’s predictions on the derivation set and further validated on the testing cohort. Two case examples of correct predictions of symptomatic and asymptomatic cases of the testing cohort were also presented, with individual contributions of each feature to the final prediction.

#### Dependency and Partial Dependency of Selected Variables

Dependence plots were used to analyze potential nonlinear, unadjusted associations between the 2 most impactful variables and their effect of symptomatic status prediction. Partial dependence plots were also examined to isolate the effect of the variables of interest while averaging out the impact of all other variables in the model. Optimal cutoffs for stratifying carotid plaques into low and high likelihood of symptomatic status were derived considering the nadir, histograms of feature distributions and normal range for the feature from the derivation set.

### ML Performance Evaluation

ML models were evaluated according to discrimination ability and calibration. The areas under both the AUROC and AUPR were constructed from ground-truth labels and predicted probabilities pooled across validation folds for internal validation, and from bootstrap samples for dedicated testing, and used as measures of discrimination. The average precision score was used to summarize the AUPR. Sensitivity and specificity were calculated at the cutoff that maximized the Youden’s J statistic (sensitivity + specificity–1) from the derivation set. Confidence bands around curves were generated with 5000 repetitions of the bootstrap on either pooled CV results (for average curves after internal validation) or testing predictions (for dedicated testing). Model calibration was assessed both qualitatively through observed versus predicted plots and quantitatively using the Brier score.^27^ The bootstrap was also used to assess predictive performance on the dedicated testing set.^27,28^ All metrics were computed using the percentile method on scores accumulated across the 100 CV repetitions for internal validation and the 5000 bootstrap resamples for the dedicated testing, providing the median and 95% CIs.

### Statistical Analysis

Continuous data were presented as mean±SD or median and interquartile rangedepending on the normality of residuals. Comparisons between symptomatic and asymptomatic subjects were performed using *t* tests or Mann-Whitney *U* tests as appropriate. Differences in proportions were assessed using the χ^2^ test. To account for multiple comparisons, *P* values were adjusted using the Benjamini-Hochberg method. Statistical significance between areas-under-curve were determined using the DeLong method or the independent *t* test, as appropriate.^29^ The net reclassification index (NRI) was employed to assess the incremental value of the proposed ML approach in comparison to traditional statistical models for improving the accurate reclassification of carotid plaques into symptomatic and asymptomatic status. Both the event-specific, nonevent specific, and the combined NRI were calculated. Cutoffs identified by our ML approach on the derivation data were further examined using both univariable and multivariable logit analysis on the testing cohort by adjusting for common clinical confounders such as demographics and cardiovascular risk factors. *P*<0.05 were deemed statistically significant. The GB-GAM model was implemented using the interpret framework in Python. All analyses were performed using R software (R Foundation for Statistical Computing, Vienna, Austria, version 4.1.0) and Python language (Python Software Foundation. Python Language Reference, version 3.9).

## RESULTS

### Characteristics of the Derivation and Testing Cohorts

**Figure 1.**
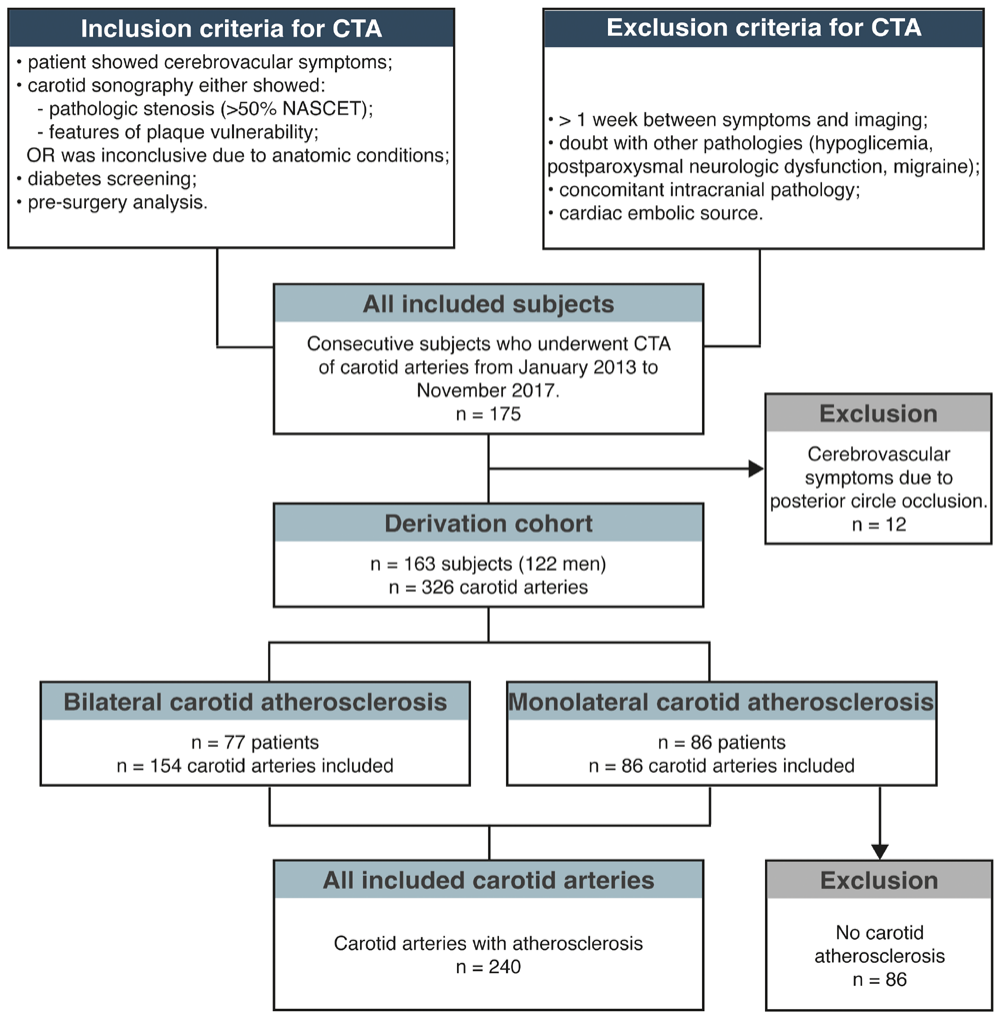
**Schematic of inclusion and exclusion of patients for computed tomography angiography (CTA) acquisition.** Details on the total number of carotid arteries constituting the derivation cohort are provided. NASCET indicates North American Symptomatic Carotid Endarterectomy Trial.

Figure 1 shows the patients flow chart. No patients were excluded due to suboptimal image quality. A total of 175 patients were enrolled during the study period. Of these, 12 were symptomatic due to posterior circle occlusion, hence they were excluded; the remaining 163 patients (122 men; median age, 72 [95% CI, 71–76]) were finally registered to the derivation cohort. Each carotid artery was evaluated independently, yielding an initial sample of 326 carotid arteries. For each subject, when both carotid arteries showed atherosclerotic plaque, the left and right sides were included (77 subjects, 154 carotid arteries). Conversely, when only one carotid artery was pathological, the normal side was excluded from the analysis (86 subjects, 86 carotid arteries). A total of 240 carotid arteries were finally used as the derivation set to train ML models. Carotid arteries in the testing cohort were exclusively used to validate the results. Patients’ characteristics in the derivation and testing cohorts are shown in Table 1.

**Table 1.**
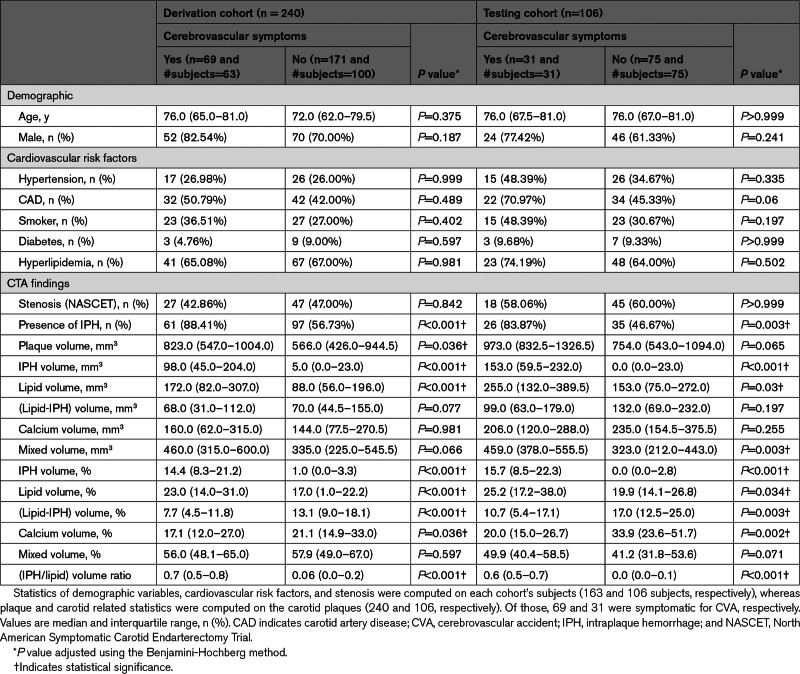
Baseline Characteristics of Patients in the Derivation and Testing Cohorts

### Diagnostic Performance in Predicting Symptomatic Plaques

**Figure 2.**
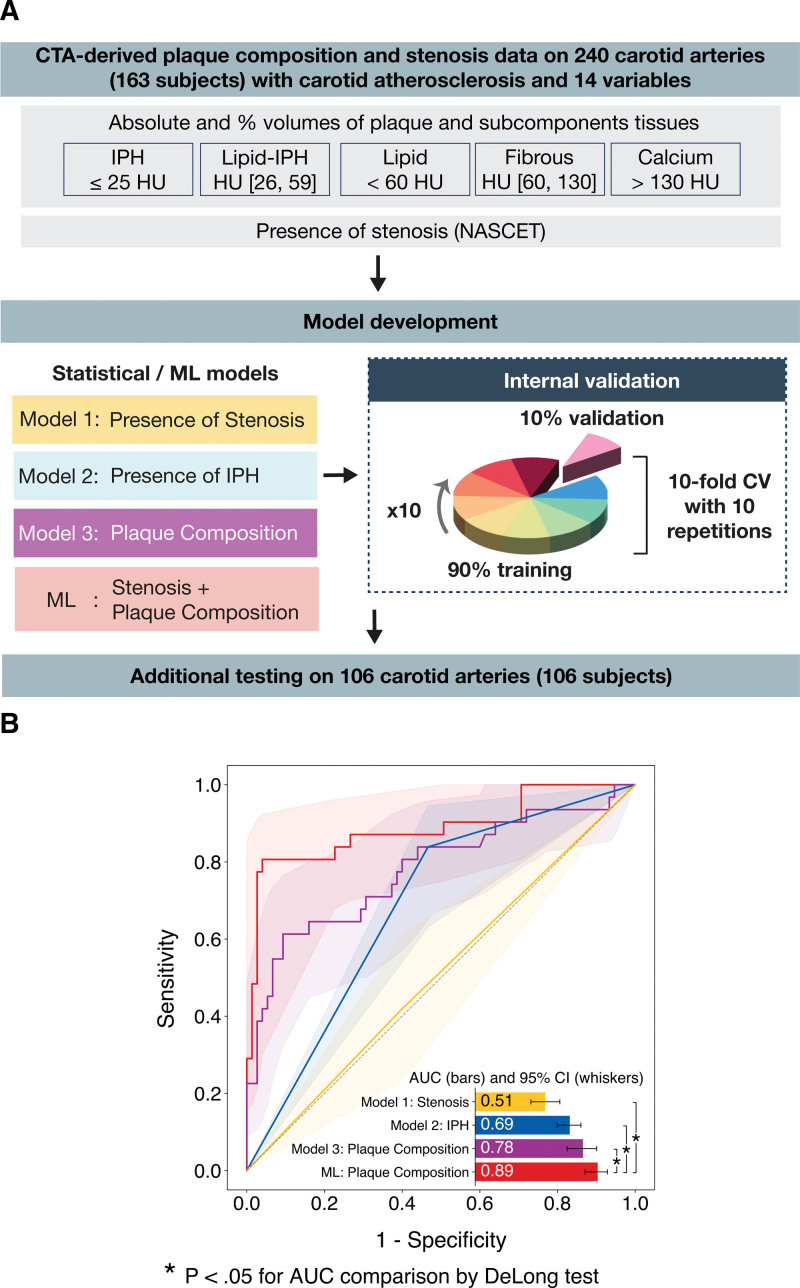
**Sequence of steps of machine learning (ML) analysis and performance of ML and traditional models. A**, ML analysis comprised model building with a tree-based boosting generalized additive model, 10 repetitions of 10-fold cross-validation (CV) for internal validation and additional testing on a dedicated test set. Four models were derived—ML-plaque composition based on plaque measures, and 3 logistic regression models, model 1: Stenosis based solely on the presence of stenosis, model 2: intraplaque hemorrhage (IPH) based on presence of IPH, model 3: plaque composition—to identify symptomatic plaques. **B**, Receiver-operating characteristics curves showing diagnostic performance of the proposed ML model and traditional logit models of stenosis, presence of IPH, and plaque components in detecting symptomatic plaques on the dedicated testing cohort. Median areas-under-curve are reported as horizontal bars with 95% CIs shown with horizontal whiskers. Comparisons between models are indicated by vertical whiskers which are annotated with asterisks to indicate statistical significance. CTA indicates computed tomography angiography; and NASCET, North American Symptomatic Carotid Endarterectomy Trial.

The overall ML process is illustrated in Figure 2A. ROC curves for identifying symptomatic plaques on the dedicated testing cohort are reported in Figure 2B. ML had the highest AUROC: 0.89 (95% CI, 0.78– 0.95) after 5000 iterations of the bootstrap at the Youden’s index identified using the derivation set (Table S3). ML achieved significantly higher areas under the curve than logistic regressors using presence of stenosis (0.51 [95% CI, 0.41–0.62]), logistic regressors using presence of IPH (0.69 [95% CI, 0.59–0.76]), and logistic regressors using plaque composition data (0.78 [95% CI, 0.65–0.87]), all *P*<0.001. ROC curves after internal validation are shown in Figure S1. ML showed a sensitivity of 81% (95% CI, 63–92) and a specificity of 95% (95% CI, 87–99; Table S3). Precision-recall curves on the testing cohort are shown in Figure S2A. AUPR of the proposed ML model was significantly higher than those of logistic regressors using presence of stenosis (0.30 [95% CI, 0.20–0.40]), logistic regressors using presence of IPH (0.40 [95% CI, 0.29–0.54]), and logistic regressors using plaque composition data (0.70 [95% CI, 0.51–0.82]), all *P*<0.001. Precision-recall curves after internal validation are reported in Figure S2B. Positive and negative predictive value were 87% (95% CI, 68–96) and 92% (95% [84–97]), respectively (Table S3).

Out of the 10 errors made by our ML model on the testing cohort, 8 (80%) were male subjects, and 2 (20%) were female subjects. Interestingly, the same ML model, when trained on a gender-balanced derivation set, exhibited an equal number of errors on both male and female subjects. This suggests that the underrepresentation of female subjects in the derivation set did not result in a biased model toward making errors on female subjects. Overall, models trained on the balanced derivation set demonstrated performance similar to those trained on the unbalanced data set. Specifically, the balanced ML model achieved the same AUROC as the unbalanced model (0.89 [95% CI, 0.79–0.95], *P*=0.88). ROC and precision-recall curves following gender balancing for all models on both dedicated testing and internal validation are provided in Figure S3. Diagnostic performance details following gender balancing are reported in Table S4.

Comparable diagnostic performance was observed on internal validation; however, these results may be overfitted and are typically not as reliable as those achieved on a dedicated testing set. The proposed ML approach achieved the highest AUROC (0.88 [95% CI, 0.66–0.99]) and AUPR (0.79 [95% CI, 0.5–0.97], all *P*<0.001), along with great calibration (0.12 [95% CI, 0.06–0.22], Figure S4A). On dedicated testing, a median Brier score of 0.09 (95% CI, 0.04–0.15) indicated excellent calibration of predicted scores (Figure S4B).

Confusion matrices, providing detailed information about correct classifications and errors for all models on the testing cohort, are presented in Figure S5. Overall, our ML approach provided incremental diagnostic value on both dedicated testing and internal validation, in reclassifying carotid plaques in symptomatic and asymptomatic status, with respect to logistic regressors using presence of stenosis (NRI of 1.21 [95% CI, 1.14–1.28] on the testing cohort, NRI of 1.29 [95% CI, 0.95–1.62] after internal validation), logistic regressors using presence of IPH (NRI of 1.13 [95% CI, 1.05–1.20] on the testing cohort, NRI of 1.24 ([95% CI, 0.89–1.58] after internal validation) and logistic regressors using plaque composition data (NRI of 1.14 [95% CI, 1.06–1.21]) on the testing cohort, NRI of 1.24 (95% CI, [0.89–1.58]), all *P*<0.001 (see Table S5 for the event- and nonevent-specific NRI).

### Importance of Variables for Identifying Symptomatic Plaques

**Figure 3.**
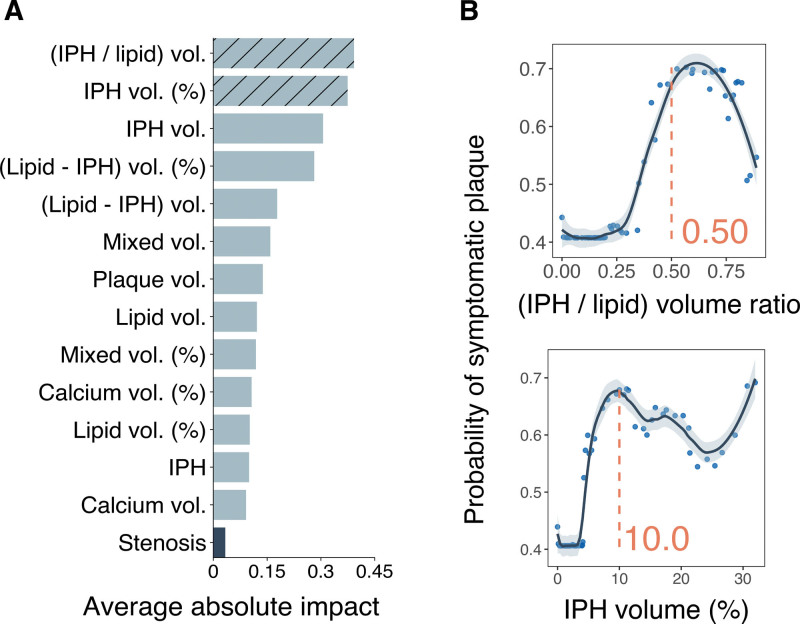
**Variable importance and associations with symptoms. A**, Variable importance and ranking for identifying symptomatic plaques from the testing set. **B**, Dependency plots showing the nonlinear relationship between the 2 most predictive plaque composition variables and the probability of symptomatic status. IPH indicates intraplaque hemorrhage.

The ML model was built using 13 CTA-derived plaque composition features and presence of stenosis as determined by ultrasound. Variable importance ranking is reported in Figure 3A.^2^ Among them, ratio of IPH to lipid volume and absolute IPH volume out of total plaque volume had the highest impact on identifying symptomatic plaques. Other plaque components such as plaque volume and lipid volume had lower impact. Presence of stenosis and presence of IPH were the weakest predictors.

### Dependency and Partial Dependency of Most Predictive Variables

Dependency plots for the 2 most predictive variables in the ML model from the derivation cohort are shown in Figure 3B. In brief, the predicted probability of plaque symptomatic status increased steadily as the ratio of IPH to lipid ratio increased, until it reached 50%, at which point it began to decrease. Similarly, as the percentage of IPH volume relative to the total plaque volume increased, there was an associated increase in the probability of symptoms until it reached 10%, after which it decreased and then increased again around 30%. Partial dependence plots that report the adjusted probability when the influence of common clinical confounders is averaged out are reported in Figure S6. Carotid plaques with ratio of IPH to lipid volume <30% exhibited the lowest likelihood of displaying symptomatic characteristics. Beyond this threshold, there was a steady increase until the 50% threshold. Similarly, values of percentage of IPH volume <5% were associated with reduced predicted probabilities, which exhibited a continuous increment until reaching the 10% cutoff, after which a declining trend was observed. Histograms of feature distributions used to identify cutoffs are presented in Figure S7.

### Individualized Explanations of ML Predictions

**Figure 4.**
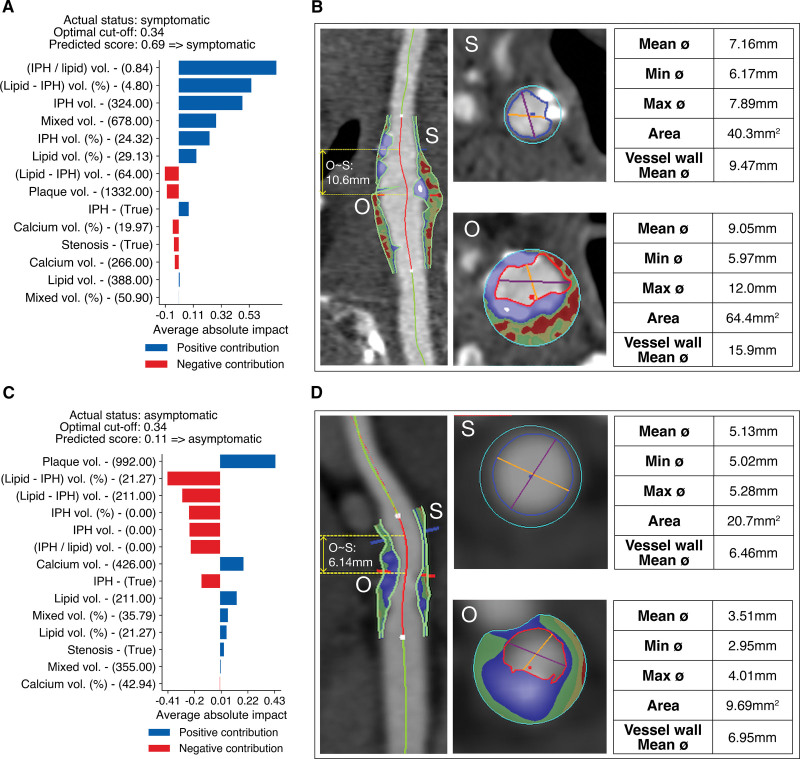
**Example of correct predictions of symptomatic and asymptomatic cases. A–C**, Personalized explanations by the machine learning model of the 2 examples of correct predictions, with individual contributions of each feature to the predicted likelihood of symptomatic status. Features are sorted decreasingly by impact; blue and red bars indicate positive and negative contributions, respectively. Gray dotted line indicates classification threshold for deciding whether the patient is symptomatic or not. **B–D**, Computed tomography angiography (CTA) view of an asymptomatic carotid plaque of a 67-year-old male and a symptomatic plaque of an 75-year-old male, respectively. IPH indicates intraplaque hemorrhage.

Examples of correct predictions by ML with individualized explanations for 2 subjects are shown in Figure 4. Individual contributions of each feature to the final predicted score along with their values are reported for both subjects.

### Validation of ML Derived Cutoffs

All ML-derived cutoffs were further examined to verify their diagnostic relevancy. Each cutoff was tested both in an univariable logit analysis and also adjusted for common clinical confounders such as age, sex, and cardiovascular risk factors. Both identified cutoffs significantly stratified carotid plaques based on the likelihood of symptomatic status. Carotid arteries exhibiting plaques with higher ratio of IPH to lipid volume (ratio IPH/lipid ≥50%; odds ratio [OR], 50.4 [95% CI, 14.4–243.4], *P*<0.001 in univariable analysis; OR, 49.2 [95% CI, 13.9–241.3], *P*<0.001 in demographics-adjusted analysis; and OR, 38.5 [95% CI, 10.1–205.1], *P*<0.001 in risk factors-adjusted analysis) and percentage of IPH volume (IPH (%) ≥10%; OR, 24.2 [95% CI, 8.3–80.5], *P*<0.001 in univariable analysis; OR, 23.5 [95% CI, 7.9–80.3], *P*<0.001 in demographics-adjusted analysis; and OR, 18.5 [95% CI, 5.7–69.4], *P*<0.001 risk factors-adjusted analysis) were significantly associated with an increased probability of symptomatic status, both on derivation and testing cohorts (Table 2 and Table S6).

**Table 2.**
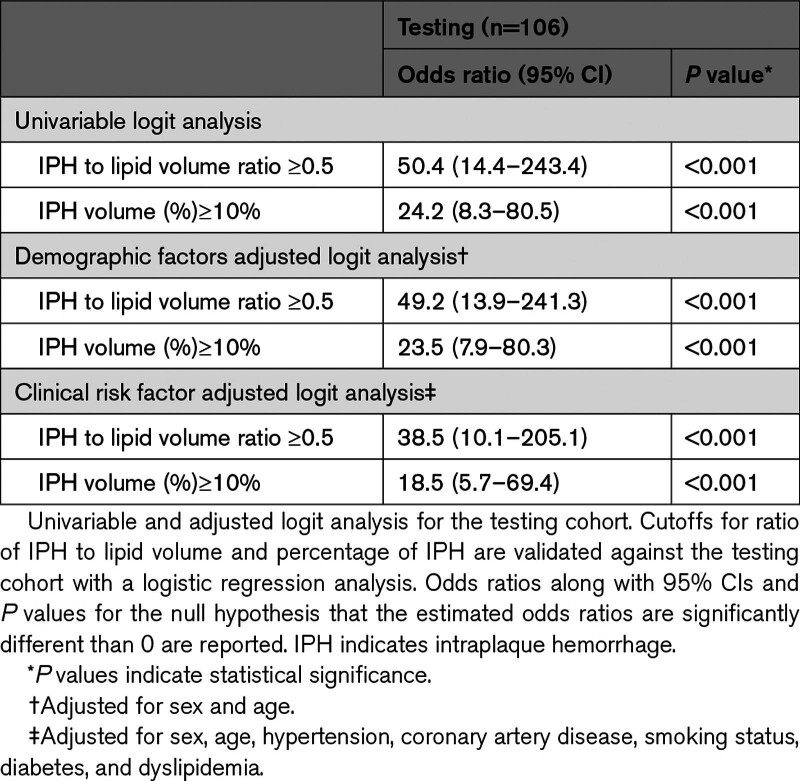
Validation of Machine Learning-Derived Cutoffs for Most Predictive Variables on the Testing Cohort

## DISCUSSION

Recent evidence has demonstrated that carotid plaque morphology correlates with ipsilateral stroke risk. Using ML, we showed that a quantitative carotid plaque component model may have significant impact on identifying symptomatic carotid plaques independent of the presence of IPH or the degree of carotid stenosis, allowing to identify symptomatic carotid plaques with optimal predictive accuracy.

The best predictors were ratio of IPH to lipid volume and percentage IPH volume and could serve as valuable imaging indicators for symptomatic carotid plaques.

From a histological perspective, a large LRNC contains an elevated concentration of free cholesterol, thereby increasing the likelihood of volume expansion through crystallization. The development and enlargement of cholesterol crystals, aligned in parallel formations with sharp tips within the necrotic core, have the potential to efficiently sever the vasa vasorum network, leading to IPH and plaque rupture.^30,31^

Indeed, histological research have reported that some morphological features of carotid plaque are related to an increased risk of cerebrovascular symptoms, including LRNC and IPH.^32^ In a recent meta-analysis, Schindler et al^33^ investigated the risk of stroke in patients with carotid artery disease with and without IPH using MRI. The presence of IPH significantly increased the risk of stroke in both symptomatic and asymptomatic patients.

In addition, plaque subcomponent volumes and percentages have shown an important role in stroke risk prediction.^34,35^

Cao et al^36^ demonstrated that prevalence, volume, and proportion of IPH were associated with the severity of stroke.

Our results are consistent with recent studies highlighting the reliability of carotid artery plaque volumetric analysis on CT as well as the importance of delineation of subcomponent volume for stroke prediction.^37,38^ In line with previous evidence, our data also suggests that the ratio and percentages of subcomponent volumes, in particular IPH and lipid volumes, are relevant for identifying symptomatic carotid plaques, suggesting that the quantification of volume and percentage of IPH may be useful in patients with carotid plaque in stroke risk stratification, beyond simply determining the presence of IPH.

The main limitations in evaluating carotid atherosclerosis using CT in clinical practice are blooming artifacts, edge blur, and halo effects. Nevertheless, the good spatial resolution of CT, the rapid flow rate achieved using a power injector, the CT scanning parameters, the applied reconstruction algorithm, and the window/level setting of 850/300, along with the expertise of neuroradiology readers, enabled us to overcome these limitations and not exclude any patients from our analysis.

AI-based models could potentially enhance stroke risk prediction, as recently suggested by Brugnara et al.^39^ The authors investigated a ML-based algorithm integrating different parameters from CT imaging to predict the clinical outcome after acute stroke reporting that the performance of a ML-based approach was higher than with conventional statistical methods. Indeed, an AI-based approach has the potential to overcome the linear association assumption made using conventional statistical analysis, allowing the examination of complex nonlinear interactions within the data.^2^

One strength of our study is that all the CTA-derived variables used to construct our ML model can be obtained on arrival at the hospital in relatively short time following a standardized protocol. Furthermore, we created an interpretable model to facilitate integration in routine clinical practice. During the training procedure, the ML model learns a contribution function for each feature, which can be then used to get the contribution to the predicted ML score; thanks to the additive nature of the ML model, contributions of all features can be summed to obtain the final score of symptomatic status. By showing example cases of ML predictions, we showed which features had the greatest impact on model predictions and individual feature contributions to the final prediction. Nonlinear relationships between the identified predictive plaque composition variables and likelihood of symptomatic status were examined and used to derive diagnostic thresholds. These thresholds were further tested, and their diagnostic relevancy were quantified with further logit analyses on the testing cohort. Our interpretable approach may offer an alternative to more complex methods which suffer from the black-box problem of explainability of outputs. Indeed, one of the challenges in the widespread applicability of AI-based models in clinical practice is their lack of interpretability, which means that the systems do not offer any insight or explanations about how the result is obtained. Currently, some AI-based tools have received approval from the Food and Drug Administration and are already being used in cardiovascular clinical practice, particularly in the field of imaging acquisition and reconstruction.^40^

However, developing AI models intelligible to human comprehension, enabling an understanding of why a model produces a specific outcome, can facilitate the widespread integration of AI algorithms into diagnostic and prognostic cardiovascular clinical settings, as well as increase the trust of physicians in AI applications.

Additionally, physicians and future medical professionals should be properly trained in the AI domain to promote the safe adoption of AI-based models in cardiovascular imaging.

There are limitations to this study. First, our findings are based on a relatively small cohort. Although we evaluated our model on a dedicated testing set, which produced^33,28^ more reliable estimates of predictive performance than internal validation, our model may benefit from additional training on bigger cohorts. In fact, the predictive performance of ML models depends, among other things, on the data set size, therefore, additional studies on bigger cohorts are needed to confirm our findings. Second, predictive performance of the final ML model was tested on testing data acquired at the same institution in a different time period than the derivation data. Although the promising results, the generalizability of the model must be further quantified on additional data with different baseline characteristics, social backgrounds, treatments and ethnicity. Additionally, although our experiments were conducted in a controlled and standardized environment, using the same scanner, scanning parameters, and reconstruction algorithm for all imaging, the generalizability of our approach requires further evaluation. Future validation studies involving multiple scanners and institutions, as well as different software tools for quantifying the volumes of plaque subcomponents, are needed to thoroughly assess the applicability of our method in diverse settings. Third, female subjects were underrepresented in both the derivation cohorts. Previous research has demonstrated convincing evidence for sex differences in carotid atherosclerotic, with plaque features of size, composition and morphology being more common or larger in man compared with women.^41^ Additionally, the incidence of stroke has been observed higher in man than women, despite risk factors being more strongly associated with the risk of any stroke in women than man.^42,43^

The diagnostic performance of both the proposed approach and traditional logistic regression models, following gender balancing, was comparable to the original models trained on data where female subjects were underrepresented, in both internal validation and dedicated testing. Furthermore, the majority of errors made on the testing cohort by our ML model, trained on the unbalanced derivation set, were associated with carotid plaques in male subjects. Interestingly, on retraining the model on a gender-balanced derivation set, the number of errors between male and female subjects remained unchanged, suggesting that the underrepresentation of females is unlikely to have introduced bias in our diagnostic model. Fourth, this was a cross-sectional study. We built ML models using data on radiological findings of a real-world cohort of patients that were symptomatic at the time of examination. Although our model can identify arteries associated with recent cerebrovascular symptoms with optimal diagnostic accuracy, additional prospective and longitudinal studies are warranted, so that more clinical covariates can be assessed, and the occurrence of future CVE can be recorded.

## CONCLUSIONS

These results add evidence to the status of intracarotid plaque subcomponent volumes and biomechanical structure in identifying subjects with recent cerebrovascular symptoms using interpretable ML. Atherosclerotic carotid plaques with a ratio of IPH to lipid volume ≥50% and a percentage of IPH volume ≥10% were associated with a higher likelihood of symptomatic status. These data-driven findings add important diagnostic information and aim to offer valuable assistance in clinical decision-making.

## ARTICLE INFORMATION

### Acknowledgments

All authors agreed with the content and gave consent to submit. All authors contributed equally as authors to this work. The authors state that this work is not under consideration elsewhere and none of the article’s contents have been previously published. The authors of this article declare no relationships with any companies, whose products or services may be related to the subject matter of the article. This research did not receive any specific grant from funding agencies in the public, commercial, or not-for-profit sectors. All authors read and approved the final article. Some of the patients under analysis were published in one of our previous studies. The scientific guarantor of this publication is the corresponding author. All authors agreed with the content and gave consent to submit.

### Sources of Funding

None.

### Disclosures

None.

### Supplemental Material

Supplemental Methods

Tables S1–S6

Figures S1–S7

## Supplementary Material
