# Supplementary material for: Machine Learning Detects Symptomatic Plaques in Patients With Carotid Atherosclerosis on CT Angiography
Source: Circ Cardiovasc Imaging. 2024 Jun 18;17(6):e016274. doi: 10.1161/CIRCIMAGING.123.016274 (PMC11186714; doi:10.1161/CIRCIMAGING.123.016274)
Supplement: Supplementary file 1 [file hci-17-e016274-s001.pdf]

SUPPLEMENTAL MATERIAL.

Supplemental Methods

Internal Validation and Dedicated Testing

Both the machine learning model and the conventional logit models were also derived using a gender-balanced derivation set. Female subjects were oversampled through the Synthetic Minority Over-sampling Technique (SMOTE) to achieve an equal representation of male and female subjects in a 1:1 ratio. SMOTE generates realistic synthetic subjects by interpolating characteristics from a restricted neighborhood of female subjects within the derivation cohort. The oversampling process was integrated into the cross-validation procedure, applied on the training subsets, and extended to the entire derivation set after selecting the best model through internal validation.

Statistical Analysis

The Brier score was used to quantitatively evaluate calibration of machine learning (ML) model predictions. The Brier score is a proper scoring method used to assess predictive performance of binary prediction models. It simultaneously assesses discrimination ability and calibration of predicted probabilities, with smaller values – closer to the 0 - indicating superior models.[21]

Supplemental Figures

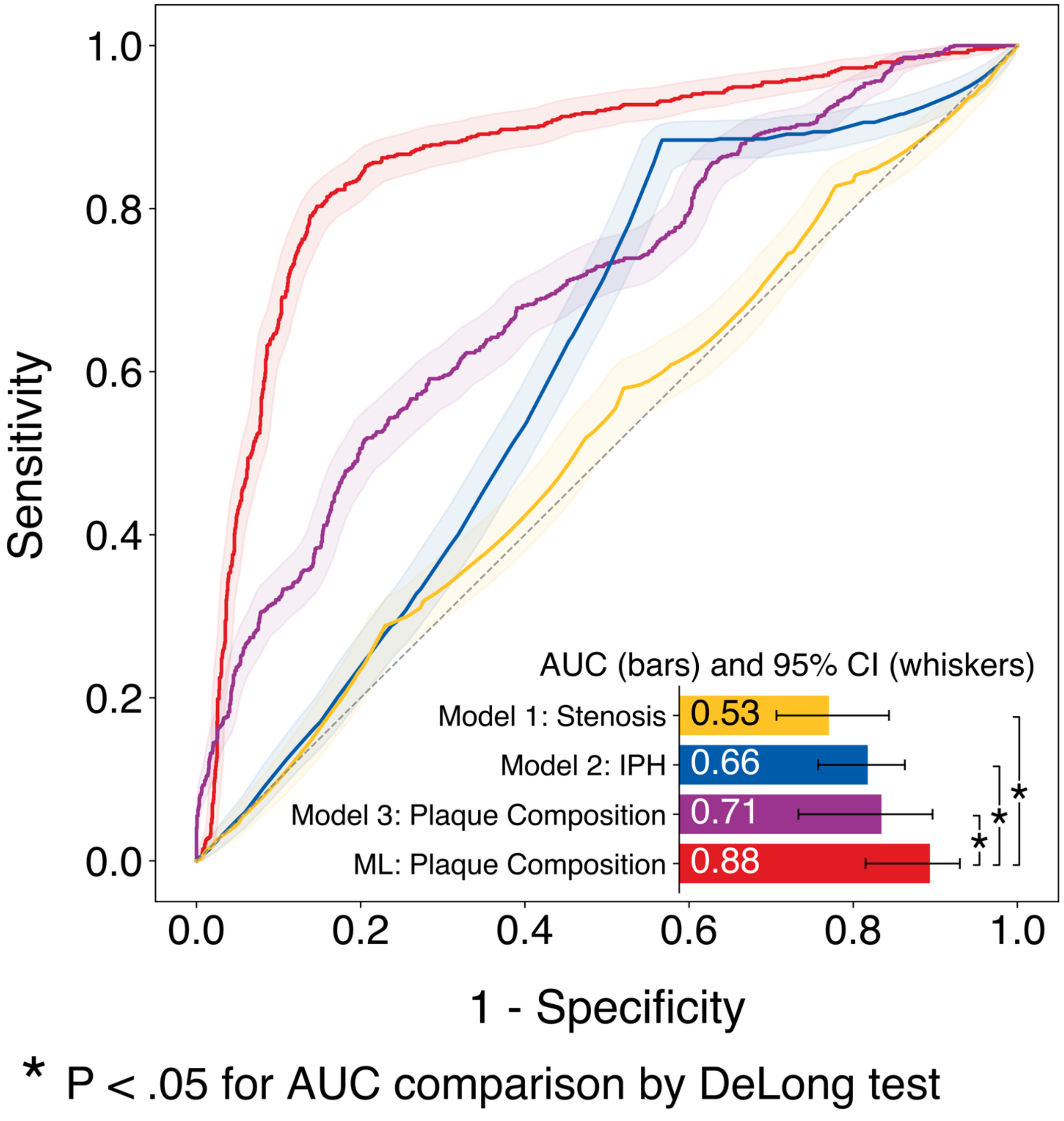

Figure S1. Diagnostic performance by receiver-operating characteristics curves on internal validation. Average receiver-operating characteristics curve after repetitions of the 10-fold testing procedure, showing diagnostic performance of the proposed ML model and traditional logit models of degree of stenosis, presence of intraplaque hemorrhage and plaque composition in detecting symptomatic plaques are shown. Median areas-under-curve are reported as horizontal bars with 95% confidence intervals shown with horizontal whiskers. Comparisons between models are indicated by vertical whiskers which are annotated with asterisks to indicate statistical significance.

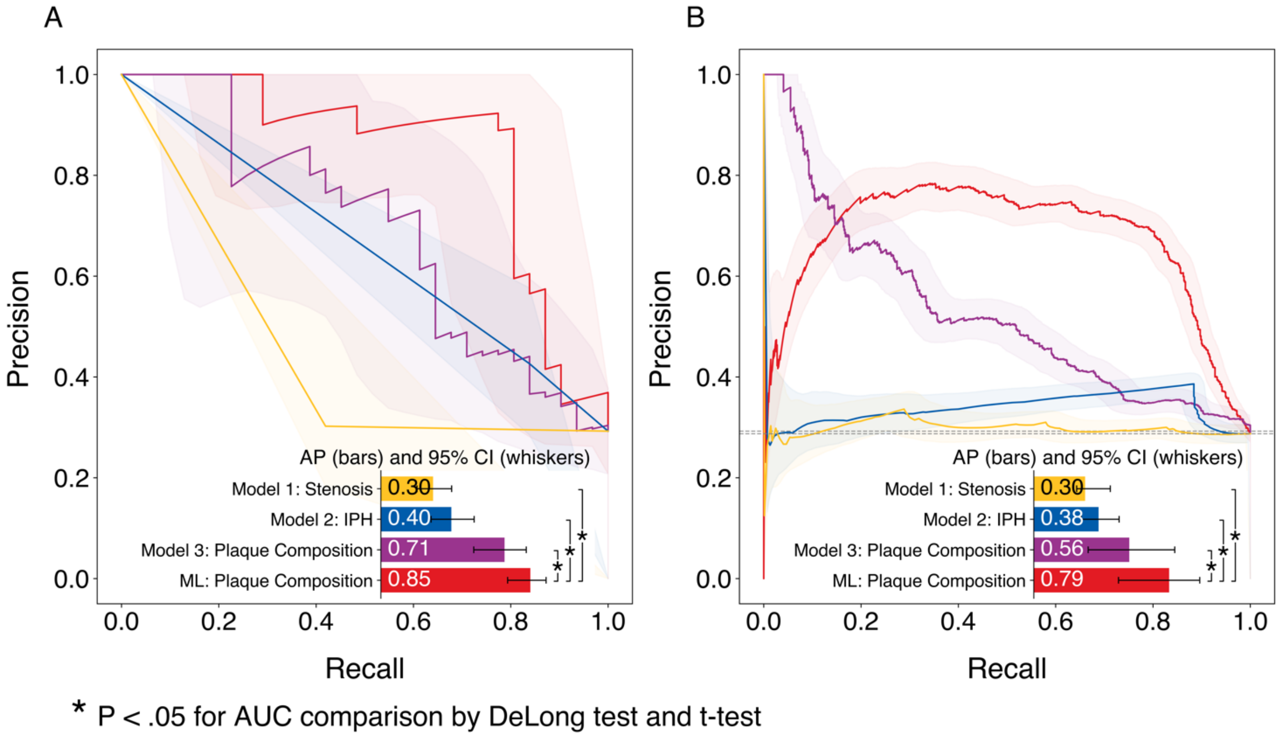

Figure S2. Diagnostic performance by precision-recall curves on dedicated testing and internal validation. A) Precision-recall curves on the dedicates testing cohort and **B**) averaged across repetitions of the 10-fold testing procedure, showing diagnostic performance of the proposed ML model and traditional logit models of degree of stenosis, presence of intraplaque hemorrhage and plaque composition in detecting symptomatic plaques are shown. Median areas-under-curve are reported as horizontal bars with 95% confidence intervals shown with horizontal whiskers. Comparisons between models are indicated by vertical whiskers which are annotated with asterisks to indicate statistical significance.

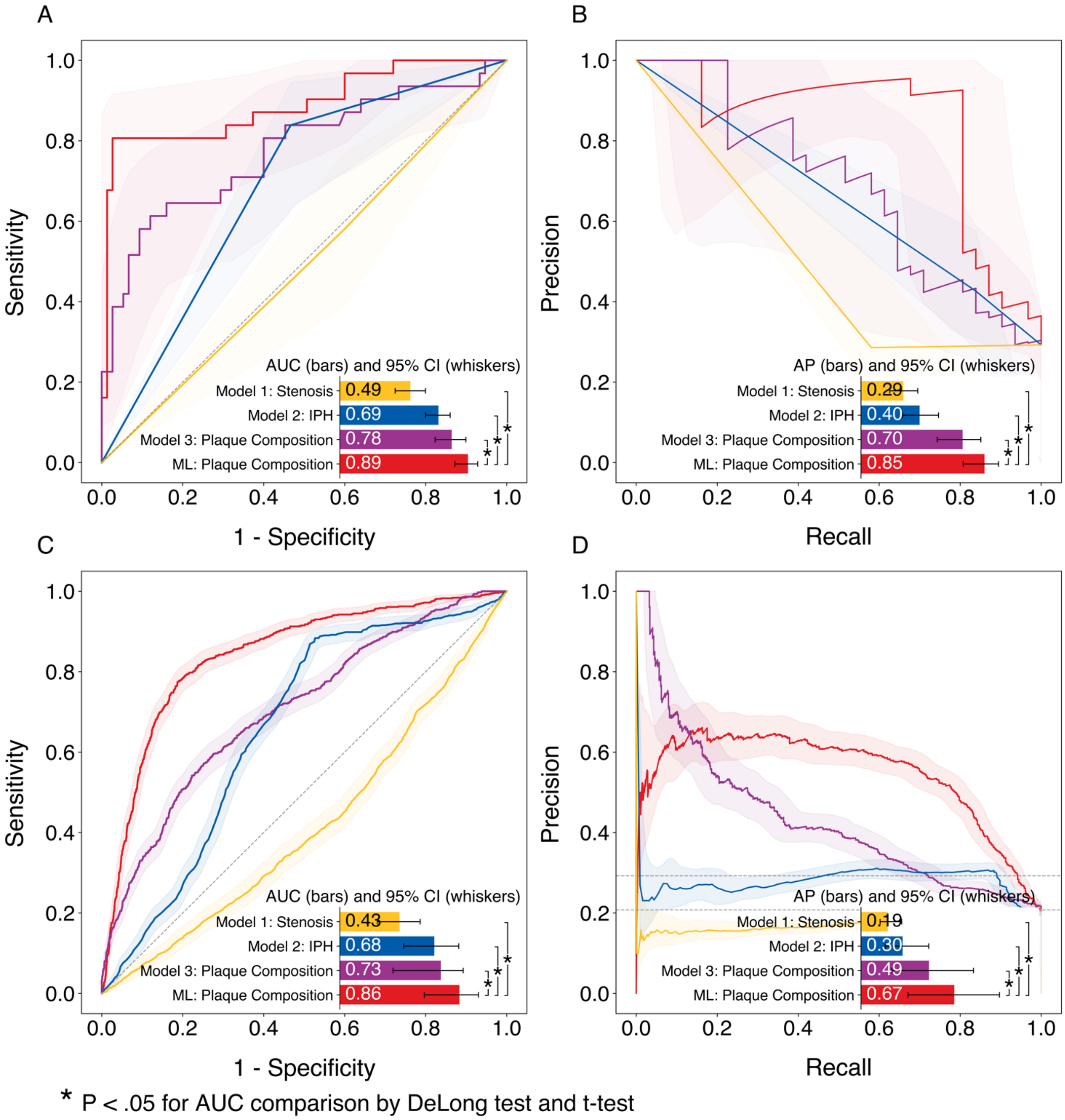

Figure S3. Diagnostic performance by receiver-operating characteristics and precision-recall curves following gender balancing on dedicated testing and internal validation. Receiver-operating characteristics and precision-recall curves on the dedicated testing cohort (**A and B**) and averaged across repetitions of the repeated 10-fold testing (**B and C**) showing diagnostic performance of the proposed ML model and traditional logit models of degree of stenosis, presence of intraplaque hemorrhage and plaque composition features in detecting symptomatic plaques are shown. Median areas-under-curve are reported as horizontal bars with 95% confidence intervals shown with horizontal whiskers. Comparisons between models are indicated by vertical whiskers which are annotated with asterisks to indicate statistical significance.

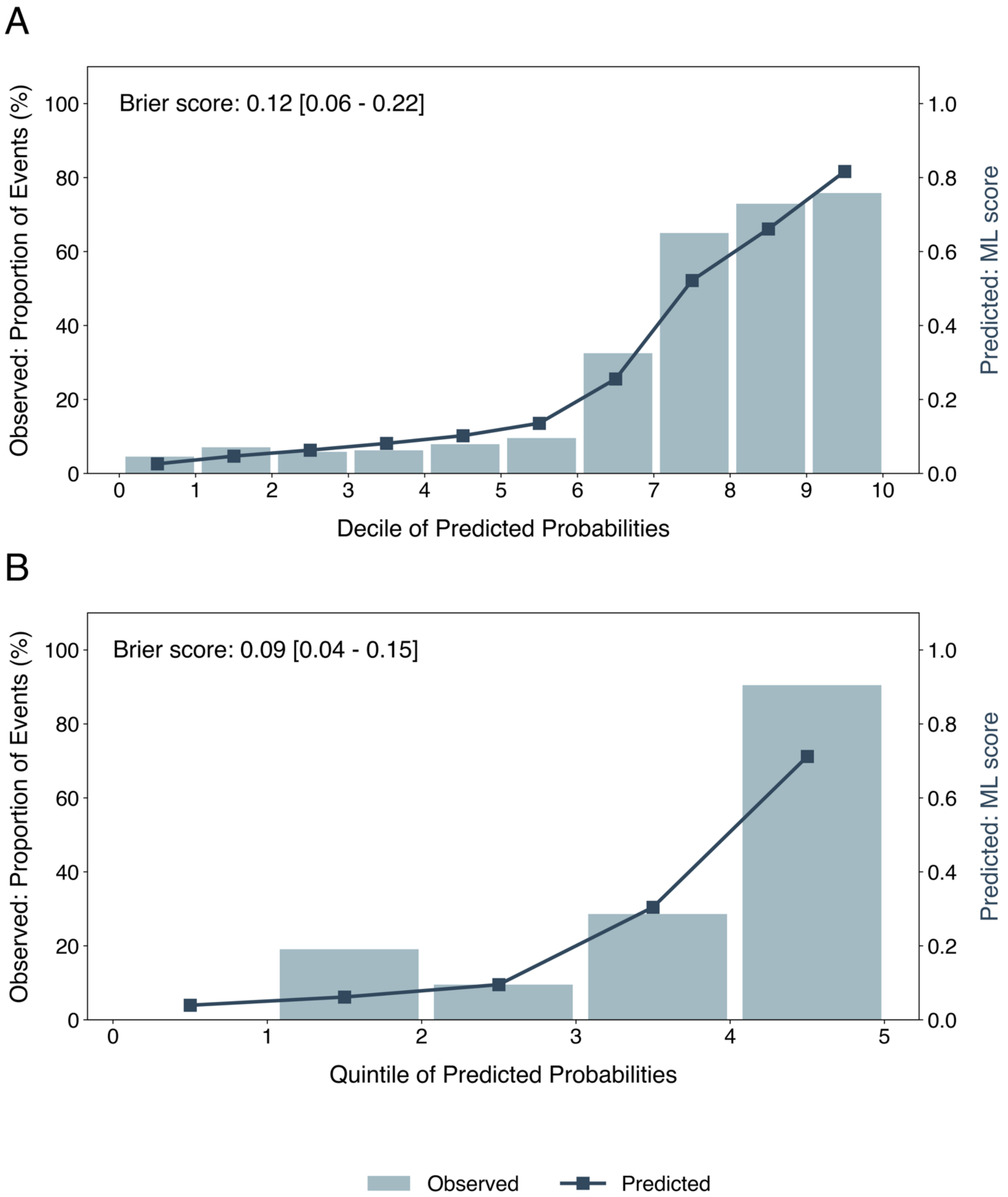

Figure S4. Calibration of the proposed ML model on both (A) derivation and (B) testing cohorts. These plots compare the observed proportion of carotid plaques associated with cerebrovascular events (vertical bars) grouped by either deciles or quintiles, with the ML-predicted score of symptomatic status (dark blue line). The median Brier score along with a 95% confidence interval is reported on the top-left corner.

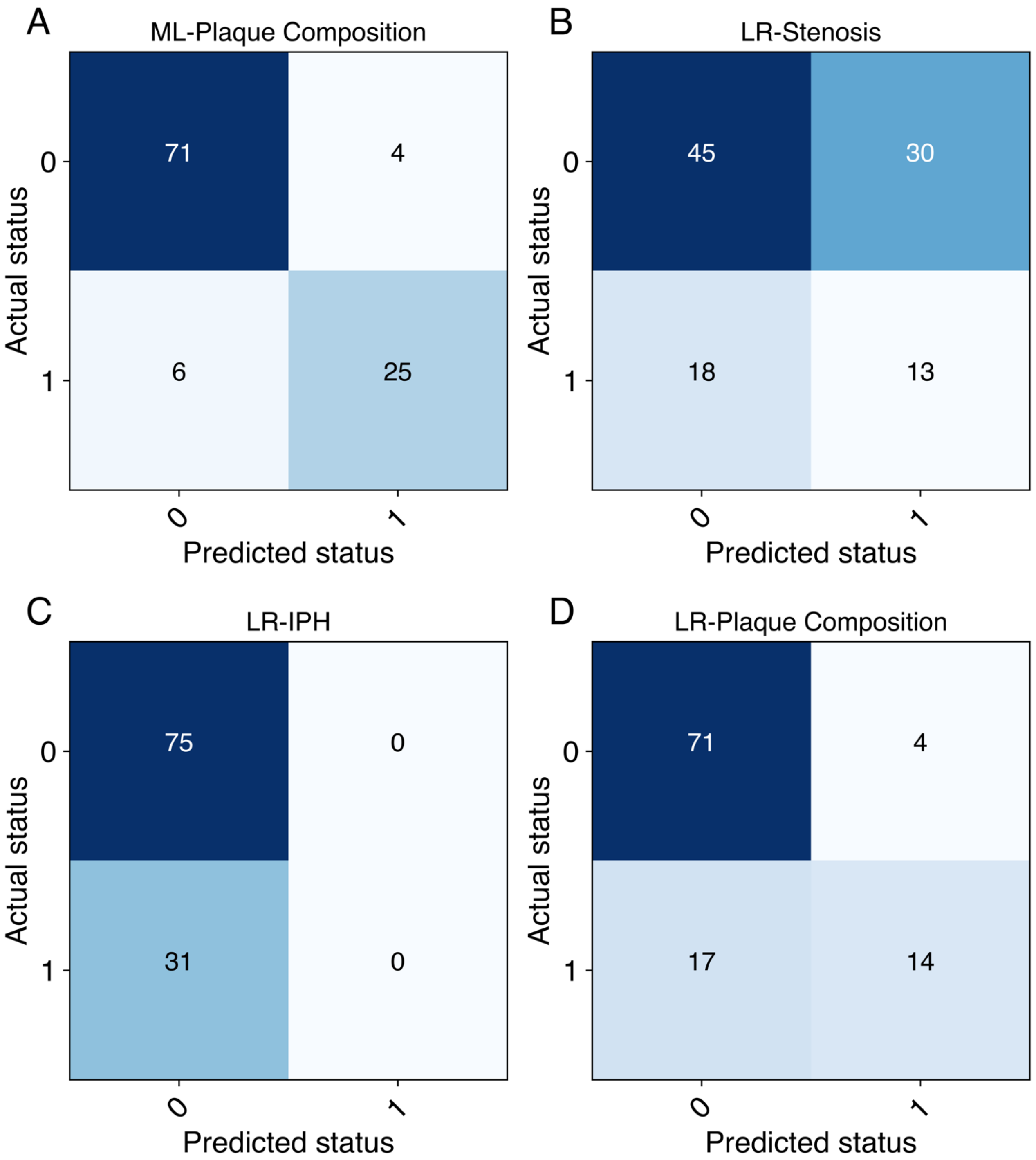

Figure S5. Confusion matrices for all models on the dedicated testing cohort.

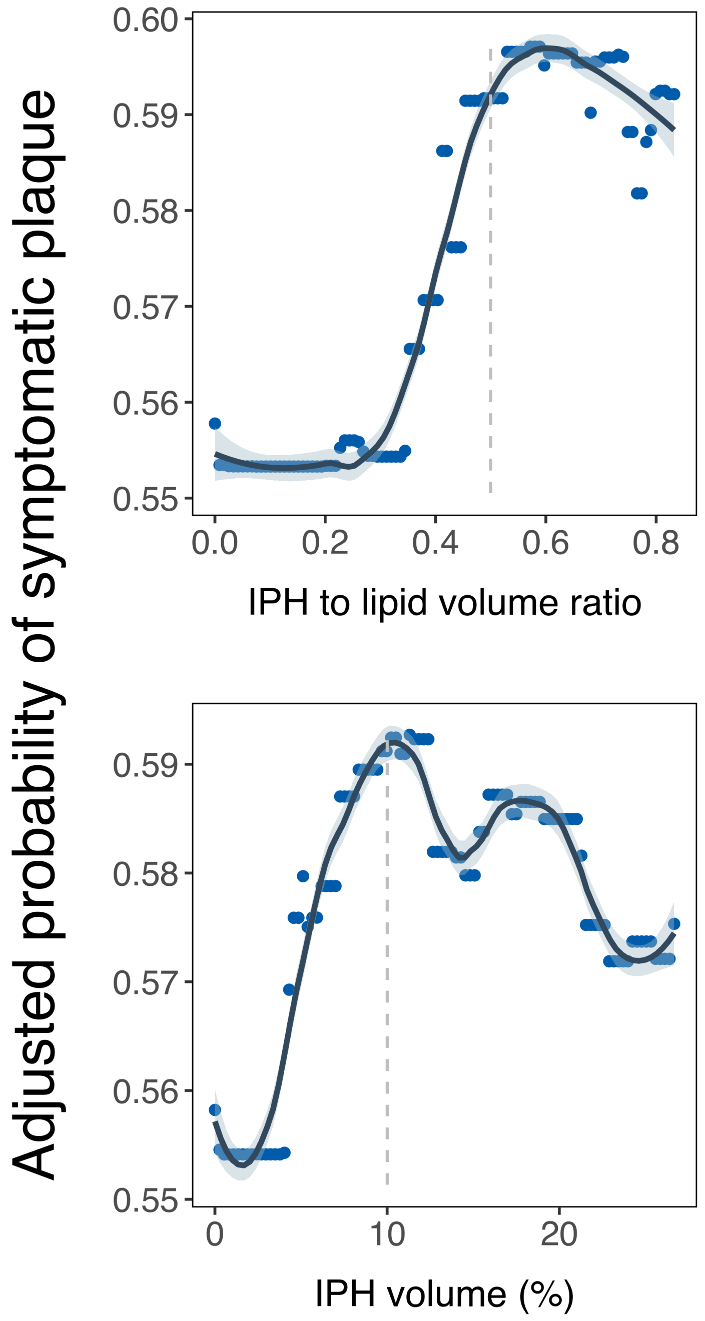

Figure S6. Partial dependency plots for the two most predictive plaque components. The non-linear relationships between variable values and predicted likelihood of plaque symptomatic status are adjusted by demographics and cardiovascular risk factors. The machine learning-derived thresholds are annotated with gray dashed lines. IPH indicates intraplaque hemorrhage.

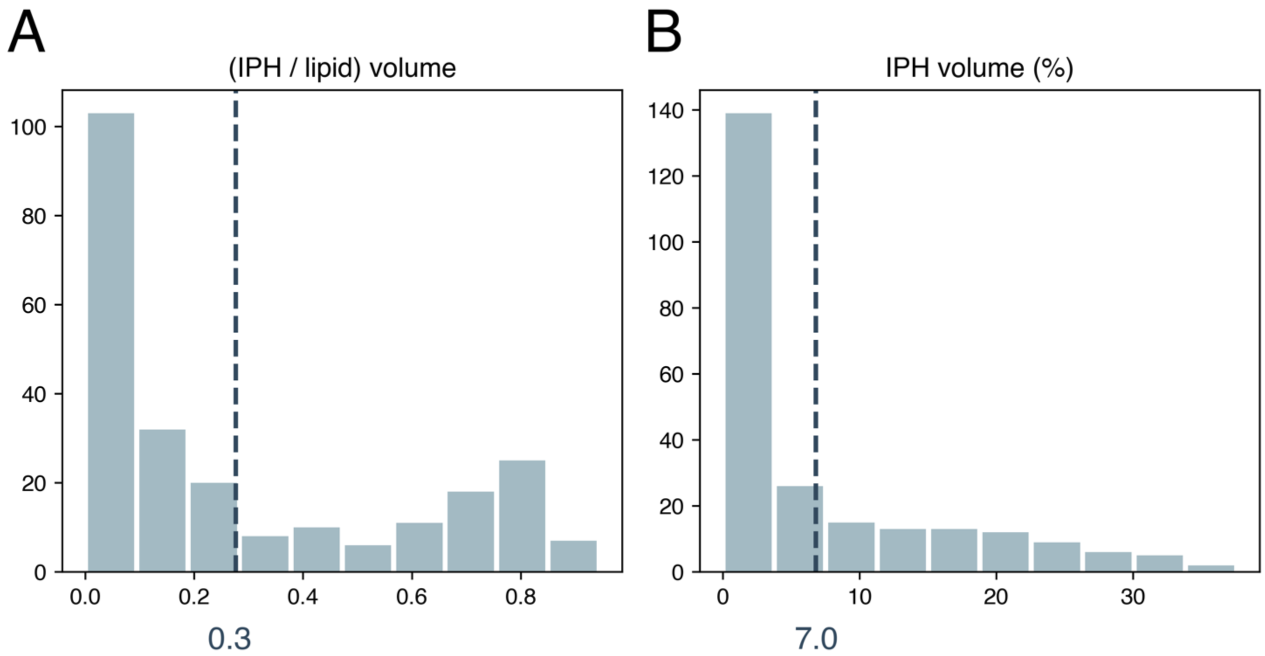

Figure S7. Histograms of the two most predictive variables in the machine learning model from the derivation cohort. Distribution of the two most predictive variables (A) ratio of intraplaque hemorrhage (IPH) and lipid volumes and (B) percentage of IPH volume out of the whole plaque's volume. Dashed lines indicate the mean value.

Supplemental Tables

Table S1. CLAIM checklist for Artificial Intelligence in Medical Imaging.

Section / Topic	No.	Item	
TITLE / ABSTRACT			
	**1**	Identification as a study of AI methodology, specifying the category of technology used (e.g., deep learning)	✓
	**2**	Structured summary of study design, methods, results, and conclusions	✓
INTRODUCTION			
	**3**	Scientific and clinical background, including the intended use and clinical role of the AI approach	✓
	**4**	Study objectives and hypotheses	✓
METHODS			
Study Design	**5**	Prospective or retrospective study	✓
	**6**	Study goal, such as model creation, exploratory study, feasibility study, non-inferiority trial	√
Data	**7**	Data sources	✓
	**8**	Eligibility criteria: how, where, and when potentially eligible participants or studies were identified (e.g., symptoms, results from previous tests, inclusion in registry, patient-care setting, location, dates)	✓
	**9**	Data pre-processing steps	✓
	**10**	Selection of data subsets, if applicable	**N/A**
	**11**	Definitions of data elements, with references to Common Data Elements	✓
	**12**	De-identification methods	**N/A**
	**13**	How missing data were handled	**N/A**
Ground Truth	**14**	Definition of ground truth reference standard, in sufficient detail to allow replication	✓
	**15**	Rationale for choosing the reference standard (if alternatives exist)	✓
	**16**	Source of ground-truth annotations; qualifications and preparation of annotators	**N/A**
	**17**	Annotation tools	**N/A**
	**18**	Measurement of inter- and intrarater variability; methods to mitigate variability and/or resolve discrepancies	✓
Data Partitions	**19**	Intended sample size and how it was determined	**N/A**
	**20**	How data were assigned to partitions; specify proportions	✓
	**21**	Level at which partitions are disjoint (e.g., image, study, patient, institution)	✓
Model	**22**	Detailed description of model, including inputs, outputs, all intermediate layers and connections	✓
	**23**	Software libraries, frameworks, and packages	✓
	**24**	Initialization of model parameters (e.g., randomization, transfer learning)	**N/A**

Training	**25**	Details of training approach, including data augmentation, hyperparameters, number of models trained	✓
	**26**	Method of selecting the final model	✓
	**27**	Ensembling techniques, if applicable	**N/A**
Evaluation	**28**	Metrics of model performance	✓
	**29**	Statistical measures of significance and uncertainty (e.g., confidence intervals)	✓
	**30**	Robustness or sensitivity analysis	✓
	**31**	Methods for explainability or interpretability (e.g., saliency maps), and how they were validated	✓
	**32**	Validation or testing on external data	✓
RESULTS			
Data	**33**	Flow of participants or cases, using a diagram to indicate inclusion and exclusion	✓
	**34**	Demographic and clinical characteristics of cases in each partition	✓
Model performance	**35**	Performance metrics for optimal model(s) on all data partitions	✓
	**36**	Estimates of diagnostic accuracy and their precision (such as 95% confidence intervals)	✓
	**37**	Failure analysis of incorrectly classified cases	✓
DISCUSSION			
	**38**	Study limitations, including potential bias, statistical uncertainty, and generalizability	✓
	**39**	Implications for practice, including the intended use and/or clinical role	✓
OTHER INFORMATION			
	**40**	Registration number and name of registry	**N/A**
	**41**	Where the full study protocol can be accessed	**N/A**
	**42**	Sources of funding and other support; role of funders	✓

Mongan J, Moy L, Kahn CE Jr. Checklist for Artificial Intelligence in Medical Imaging (CLAIM): a guide for authors and reviewers. Radiol Artif Intell 2020; 2(2):e200029. https://doi.org/10.1148/ryai.2020200029

Table S2. Description of variables used in machine learning. CAD indicates coronary heart disease; IPH, intraplaque hemorrhage.

**Name**	**Values**	**Description**
**Imaging findings**		
Stenosis	Continuous; %	Percentage of stenosis (NASCET criteria)
IPH	Continuous; mm^3	Does the plaque have intraplaque hemorrhage
Plaque volume	Continuous; mm^3	Total volume of carotid plaque
Lipid volume	Continuous; mm^3	Volume of lipid tissue subcomponent
Mixed volume	Continuous; mm^3	Volume of mixed tissue subcomponent
Calcium volume	Continuous; mm^3	Volume of calcium tissue subcomponent
IPH volume	Continuous; mm^3	Volume of IPH subcomponent
Lipid-IPH volume	Continuous; mm^3	Volume of IPH-lipid tissue subcomponent
% of lipid volume	Continuous; %	Percentage of lipid subcomponent's volume
% of mixed volume	Continuous; %	Percentage of mixed subcomponent's volume
% of calcium volume	Continuous; %	Percentage of calcium subcomponent's volume
% of IPH volume	Continuous; %	Percentage of IPH subcomponent's volume
% of lipid-IPH volume	Continuous; %	Percentage of lipid-IPH subcomponent's volume
IPH to lipid volume ratio	Continuous	Ratio of IPH and lipid subcomponents' volumes
**Target**		
Symptoms	Binary; 0/1	Whether the patient experienced cerebrovascular symptoms

Table S3. Predictive performance of the proposed machine learning model on both internal validation and on the dedicated testing set. For each evaluation metric the median value along with a 95% confidence interval is reported.

	**Internal cross-validation**	**Dedicated Testing**
**Sensitivity**	86% [43 - 100]	81% [63 - 92]
**Specificity**	88% [71 - 100]	95% [87 - 99]
**Positive predictive value**	70% [47 - 100]	87% [68 - 96]
**Negative predictive value**	93% [78 - 100]	92% [84 - 97]
**F1 score**	0.75 [0.43 - 92]	0.83 [0.70 - 0.92]
**Area under the ROC curve**	0.88 [0.66 - 0.99]	0.89 [0.78 - 0.95]
**Area under the PR curve**	0.79 [0.5 - 0.97]	0.85 [0.70 - 0.93]
**Brier score**	0.12 [0.06 - 0.22]	0.09 [0.04 - 0.15]

Table S4. Predictive performance of the proposed machine learning model on both internal validation and on the dedicated testing set following gender balancing. For each evaluation metric the median value along with a 95% confidence interval is reported.

	**Internal cross-validation**	**Dedicated Testing**
**Sensitivity**	86% [38 - 100]	81% [63 - 92]
**Specificity**	80% [57 - 99]	92% [83 - 96]
**Positive predictive value**	50% [26 - 92]	81% [62 - 92]
**Negative predictive value**	94% [82 - 100]	92% [84 - 97]
**F1 score**	0.62 [0.36 – 0.85]	0.81 [0.68 - 0.90]
**Area under the ROC curve**	0.86 [0.61 – 1.0]	0.89 [0.79 - 0.95]
**Area under the PR curve**	0.67 [0.34 - 0.99]	0.85 [0.66 - 0.93]
**Brier score**	0.11 [0.05 - 0.2]	0.11 [0.06 - 0.18]

Table S5. Net reclassification indexes of the proposed approach versus traditional statistical models. P values for statistical significance are reported in parenthesis. LR indicates logistic regression; ML, machine learning; NRI net reclassification index.

	**Internal validation**					
	**Events**		**Non-events**		**Combined**	
	NRI (95% CI)	P	NRI (95% CI)	P	NRI (95% CI)	P
**ML-Plaque Composition vs:**						
**LR-Stenosis**	0.49 (0.42-0.55)	< .001	0.72 (0.69-0.75)	< .001	1.21 (1.14–1.28)	< .001
**LR-IPH**	0.33 (0.26-0.40)	< .001	0.8 (0.77-0.83)	< .001	1.13 (1.05–1.20)	< .001
**LR-Plaque Composition**	0.34 (0.27-0.41)	< .001	0.8 (0.77-0.82)	< .001	1.14 (1.06–1.21)	< .001
	**Dedicated testing**					
	**Events**		**Non-events**		**Combined**	
	NRI (95% CI)	P	NRI (95% CI)	P	NRI (95% CI)	P
**ML-Plaque Composition vs:**						
**LR-Stenosis**	0.42 (0.1-0.74)	.01	0.87 (0.75-0.98)	< .001	1.29 (0.95–1.62)	< .001
**LR-IPH**	0.29 (-0.05-0.63)	.09	0.95 (0.87-1.02)	< .001	1.24 (0.89–1.58)	< .001
**LR-Plaque Composition**	0.29 (-0.05-0.63)	.09	0.95 (0.87-1.02)	< .001	1.24 (0.89–1.58)	< .001

Table S6. Validation of machine learning-derived cut-offs for most predictive variables on the derivation cohort. Univariable and adjusted logit analysis for the derivation cohort. Cut-offs for ratio of intraplaque hemorrhage (IPH) to lipid volume and percentage of IPH are validated against the testing cohort with a logistic regression analysis. Odds ratios along with 95% confidence intervals and P-values for the null hypothesis that the estimated odds ratios are significantly different than 0 are reported. IPH indicates intraplaque hemorrhage.

	**Derivation (n=240)**	
	**Odds ratio (95% CI)**	**P-value[a]**
**Univariable logit analysis**		
IPH to lipid volume ratio ≥ 0.5	27.4 (13.3 - 59.7)	**<0.001**
IPH volume (%) ≥ 10%	18.3 (9.3 - 37.8)	**<0.001**
**Demographic factor-adjusted logit analysis[b]**		
IPH to lipid volume ratio ≥ 0.5	27.8 (13.3 - 61.5)	**<0.001**
IPH volume (%) ≥ 10%	18.5 (9.3 - 38.5)	**<0.001**
**Clinical risk factor-adjusted logit analysis[c]**		
IPH to lipid volume ratio ≥ 0.5	31.1 (14.4 - 72.6)	**<0.001**
IPH volume (%) ≥ 10%	19.4 (9.5 - 41.9)	**<0.001**

[a]Bold P-values indicate statistical significance.

[b]Adjusted for sex and age.

[c]Adjusted for hypertension, CAD, smoking status, diabetes and dyslipidemia
